# Temporal Aspects of Surface Water Quality Variation Using Robust Statistical Tools

**DOI:** 10.1100/2012/294540

**Published:** 2012-07-31

**Authors:** Adamu Mustapha, Ahmad Zaharin Aris, Mohammad Firuz Ramli, Hafizan Juahir

**Affiliations:** Centre of Excellence for Environmental Forensics, Faculty of Environmental Studies, Universiti Putra Malaysia, 43400 UPM Serdang, Selangor, Malaysia

## Abstract

Robust statistical tools were applied on the water quality datasets with the aim of determining the most significance parameters and their contribution towards temporal water quality variation. Surface water samples were collected from four different sampling points during dry and wet seasons and analyzed for their physicochemical constituents. Discriminant analysis (DA) provided better results with great discriminatory ability by using five parameters with (*P* < 0.05) for dry season affording more than 96% correct assignation and used five and six parameters for forward and backward stepwise in wet season data with *P*-value (*P* < 0.05) affording 68.20% and 82%, respectively. Partial correlation results revealed that there are strong (*r*_*p*_ = 0.829) and moderate (*r*_*p*_ = 0.614) relationships between five-day biochemical oxygen demand (BOD_5_) and chemical oxygen demand (COD), total solids (TS) and dissolved solids (DS) controlling for the linear effect of nitrogen in the form of ammonia (NH_3_) and conductivity for dry and wet seasons, respectively. Multiple linear regression identified the contribution of each variable with significant values *r* = 0.988, *R* ^2^ = 0.976 and *r* = 0.970, *R* ^2^ = 0.942 (*P* < 0.05) for dry and wet seasons, respectively. Repeated measure *t*-test confirmed that the surface water quality varies significantly between the seasons with significant value *P* < 0.05.

## 1. Introduction

River water represents a readily available source of water for human activities and historically many civilizations have relied on the ample supplies of fresh water found in major river catchment. Currently, rivers worldwide serve as the recipient of great quantities of waste discharge by agricultural, industrial, and domestic activities [1]. The availability of fresh water in rivers is one of the major issues facing the human population especially in developing countries [2]. The constant discharge of domestic and industrial wastewater and seasonal surface run-off due to the climate change all have a strong effect on the river discharge and water quality [3]. Information on water quality and pollution sources is important for the implementation of sustainable water resource management strategies [4]. Physical and chemical characterization of aquatic environment has become an important aspect due to the seasonality of river water [5].

High concentrations of all kinds of pollutants have an influence on the river water quality and determine the use of water and also can lead to diverse problems such as algal blooms, loss of oxygen, and loss of biodiversity [6]. It is, therefore, necessary to monitor river water quality, understand the chemistry of the water, and provide a reliable assessment of water quality for effective water resource management.

In modern research, different statistical techniques such as multivariate statistical analysis through principal component analysis, cluster analysis, discriminant analysis, and multiple linear regressions have been used to evaluate and interpret complex datasets to better understand the river water quality [3]. Statistical tools have often been used in exploratory data analyses for classification of sampling stations [7, 8], identification of possible pollution sources [9–14], and identifying common patterns in data distribution that allow identification of the most significant variable responsible for river water variation [9, 15–18]. Very recently, statistical approaches have been applied to the data observed in several complex systems where the problem of environmental data reduction and interpretation can be easily handled through the application of robust statistical techniques. These statistical analyses are capable of allowing the detection of long-range correlations that are artificial nonstationeries compared to traditional conventional methods. Conventionally, the usual methods of interpretation of surface water quality are only descriptive and lack statistical significance. Furthermore, it is only relied on univariate procedure, which is inadequate to characterize simultaneous similarities and differences between samples and variables in a complex environment, hence the need to apply robust statistical tools to the surface water quality datasets. Several researchers apply robust statistical tools to evaluate surface water quality variation. For example, a study conducted by Koklu et al. [15] revealed that DA gave indicator parameters responsible for large variation in water quality and multiple regressions analysis identified the important and effective parameters that contributed to water quality variation in Melen River system, Turkey. A recent study conducted by Osman et al. [19] found that DA is an important multivariate statistical tool that reduces dimensionality of the data and brings out the most statistically significant parameters that result in variation of the datasets. Zhang et al. [6] used DA to evaluate water quality variation in southwest new territories and Kowloon, Hong Kong. They concluded that DA provided an important data reduction by revealing only four and eight parameters with 84.2% and 96.1% correct assignment for temporal and spatial water quality variation, respectively.

Jakara Basin is located in the northwestern Nigeria and lies in the center of Kano city, the most populous city in the whole of Nigeria with over six million people. The region has rapid population growth and industrial development, which increase the mass of sewage discharge. With an increase in population, surface water quality needs to be monitored continuously in order to take measures, when necessary to sustain the portability of the surface water resources [20]. Jakara Basin is located on longitude 8°31′E to 8°45′ and latitude 12°10′N and 12°13′N. The basin is about 30 km^2^ with northwest, southwest orientation sprawling about 0.33°. The climate of the area is strongly influenced by the tropical maritime air masses during wet season and tropical continental air masses during dry season. The seasonal migration of the intertropical discontinuity (ITD) gives rise to two seasons, one dry and the other wet. The wet season lasts from June to September although May is sometimes humid. The dry season extends properly from mid-October of one calendar year to mid-May of the next. The annual mean rainfall in the region is between 800 mm and 900 mm.Variation of the mean value is up to +30 or −30 percent. More than 300 mm of the rainfall is received in August alone, while the truly wet season lasts from June to September. In addition, the mean monthly temperature of the study area is 21°C and 23°C with diurnal range of 12–14°C [17].

The present study aims at evaluating the temporal variation of river water quality and determining the most meaningful parameters and their contribution towards water quality variation between dry and wet seasons in Jakara Basin.

## 2. Material and Methods

### 2.1. Sample Collection and Analytical Technique

Samplings were carried out every day from 1st April to 31st May, 2011 and 31st July to 30th September, 2011 for dry and wet seasons, respectively, at four different sampling locations along Jakara River. Samples were taken from 10 cm to 15 cm below the surface water using acid washed plastic container to avoid unpredicted changes. Samples were stored in a chilled cold box during transportation to the laboratory. Fifteen physicochemical water quality parameters were selected for analyses, these being dissolved oxygen (DO), five-day biochemical oxygen demand (BOD_5_), chemical oxygen demand (COD), suspended solids (SS), pH, conductivity, salinity, temperature, nitrogen in the form of ammonia (NH_3_), turbidity, dissolved solids (DS), total solids (TS), nitrates (NO_3_), chloride (Cl), and phosphates (PO_4_). Samples were analyzed in the Soil and Water Laboratory of Ministry of Environment, Kano, Nigeria.

The samples were filtered using filter paper with a pore size of 5 *μ*m [21]. Water temperature, DO, pH, conductivity and turbidity of the water samples were determined and detected using multiparameters monitoring instrument (YSI incorporated, Yellow Spring OH, USA). The instruments were calibrated using specific calibrating solutions. A mean value was calculated for each parameter, with standard deviation (SD) being used as an indication of the precision of each parameter [18]. NH_3_ was measured using ultraviolet absorbance spectrophotometer. The UV light absorbance NH_3_ analyzer was calibrated to measure the wavelength of UV light (within the range of 200–450 nm). NaOH reagent was added to the sample to act as a buffer by adjusting the pH of the sample to a value greater than 12. Second reagent hypochlorite was added to react with free NH_3_ in the samples to form monochloramine. The difference in the UV light is proportional to the amount of free NH_3_ in the sample. TS was measured by drying the sample at temperature of 105°C in preweighted porcelain and then cooled in a dry atmosphere in desiccators and then weighted on an analytical balance by subtracting the porcelain dish and dividing by the original amount of sample. DS was measured by filtering the water sample through a tarred fiber filter, which was then dried and the weight of the materials captured on the filter was used to figure the total suspended solids (TSS). The DS can be estimated from the difference between the TS and TSS. BOD determination of the water samples was carried out using the standard method [22]. The dissolved oxygen content was determined before and after the incubation. Sample incubation was for 5 days at 20°C in BOD bottle and BOD_5_ was calculated after the incubation period. COD was determined after oxidation of organic matter in strong tetraoxosulphate VI acid medium by K_2_Cr_2_O_7_ at 148°C with back titrations. Cl was determined using 100 mL of the water sample, which was measured into 250 mL conical flask, and pH was adjusted with 1 M NaOH. 1 mL K_2_Cr_2_O_4_ indicator was then added and titrated with AgNO_3_ solution. A blank titration was carried out using distilled water and Cl in mg/L was then calculated. NO_3_ and PO_4_ were determined using calorimetric method [22]. 

### 2.2. Data Management and Treatment

The normality distribution test of the data for each variable under study was checked by analyzing statistical value of kurtosis and skewness. The original data showed that the value of kurtosis ranged from −0.37 to 54.68 and from −0.29 to 14212, and the skewness value ranged from −0.29 to 6.93 and from −0.02 to 3.73 for both dry and wet seasons data, respectively, indicating that the data were not normally distributed.

The raw data of all the parameters under study were log transformed *x* = log_10_(*x*). Log transformation removes outliers and renders geochemical data normalized. Although log transformation is generally used to obtain normal distribution, it can also be applied to standardize the datasets and reduce the influence of extreme cases and outliers [23–25]. After the transformation the kurtosis ranged from −1.42 to 7.08 and from −0.75 to 6.52, and the value for skewness ranged from −2.50 to 2.06 and from −2.39 to 1.20 for both dry and wet seasons data, respectively. These ranges showed that both data were now within the normal distribution population.

### 2.3. Statistical Analysis

#### 2.3.1. Discriminant Analysis (DA)

Discriminant analysis is a statistical method which determines the variables that discriminate between two or more naturally occurring groups [16]. It constructs a discriminate function (DF) for each group as in equation

(1) f(Gi)=ki+∑(i=1)n(wi−qi),

where *i* is the number of groups (*G*), *ki* is the constant inherit to each group, *n* is the number of parameters used to classify set of data into a given group, and *w* is the weight coefficient assigned by DA to a given selected parameter *q*.

 In this study, temporal (dry and wet seasons) data were evaluated. DA was applied to the log transformed data using the standard, forward stepwise, and backward stepwise modes and construct DFs to evaluate temporal variation in river water quality.

#### 2.3.2. Partial Correlation

Partial correlation allows looking at the relationship between bivariates when the effect of the third variable is held constant. Partial correlation is similar to Pearson's product moment correlation except that it also allows control for an additional variable. This is usually the variable that you suspect might be influencing the two variables of interest [24, 26].

#### 2.3.3. Multiple Linear Regression

Multiple linear regression is a statistical tool for understanding the relationship between an outcome variable and several predictors (independent variables) that best represent the relationship in a population [15]. The technique is used for both predictive and explanatory purposes within experimental and nonexperimental designs. Multiple linear regressions can be expressed using the equation:

(2) Y=βo+β1X1+β2X2+⋯+βmXm+ε,

where *Y* represents the dependent variable, *X*1 ⋯ *Xm* represent the several independent variables, *βo* ⋯ *βm* represent the regression coefficients, and *ε* represents the random error.

#### 2.3.4. Repeated Measure Sample *t*-Test

This statistical tool performs a paired two-sample *t*-test to deduce whether the difference between the sample means is statistically distinct from a hypothesized difference. Repeated measure test does not assume that the variances of both populations are equal, it is used when only one group of experiment and data is collected from two different occasions or under two different conditions [26]. The *t*-value result from the analyses ranges between − infinity and + infinity, in which positive value indicates an increase while negative value indicates a decrease. The repeated measure test is calculated using equation

(3) t=X−1−X−2sP2/n1+sP2/n2,

where *t* is the test statistic (Student's *t*-distribution) ${\overset{-}{X}}_{2}$ is the mean of the paired difference for the sample ${\overset{-}{X}}_{2}$ is the mean of the paired difference for the population *s*_*P*_^2^ is the standard error of the mean of the paired difference for the sample *n*_1_ is the number of paired difference values, and *n*_2_ is the number of paired difference values

## 3. Results and Discussion

### 3.1. Descriptive Statistics

The descriptive statistics of physiochemical parameters under study are given in Table 1. It provides a summary of the mean, standard deviation, variance, sekwness, and kurtosis values of fifteen measured parameters for both dry and wet seasons data. The pH value of the water samples is acidic to slightly above neutral ranging from 6.67 to 7.14 for dry and wet seasons, respectively.

The mean for temperature and conductivity ranged from 28°C and 1.96 *μ*S/cm to 29.5°C and 4.10 *μ*S/cm for dry and wet seasons, respectively. The values of DS, TS, SS, turbidity, and salinity are generally more enhanced in wet season. These parameters are reactive compounds and qualitatively reflect the status of inorganic pollution, dissolved solids increases salinity as well as conductivity measures. The reason for the high values of these parameters during wet season could have been the result of the geology of the area and soil erosion effects. 

The mean values of DO, BOD_5_, and COD are more pronounced in dry season than wet season. This represents organic and nutrients pollution and may be from natural organic matter decomposition. This suggests that during dry season, the volume of the water in the river significantly reduced and there is substantial addition of organic materials from residential areas of Kano Metropolitan to the Jakara River [27].

### 3.2. Discriminant Functions

 The objective of DA was to test the significance of discriminant functions and to determine the most significance variables that result in water quality variation in both dry and wet seasons.

Tables 2 and 3 show that the values of Wilks' lambda for both dry and wet seasons for each discriminant function were quite small (0.37, 0.42, 0.42: dry season and 0.25, 0.84, 0.48: wet season) for standard, forward stepwise and backward stepwise mode-respectively. In the forward stepwise mode, variables/parameters were included step by step, beginning with the most significant variable until no significant changes were obtained. In the backward stepwise mode, variables/parameters were removed step by step beginning with the least significant variable until no significant changes were obtained [4, 15, 16].

**Figure 1:**
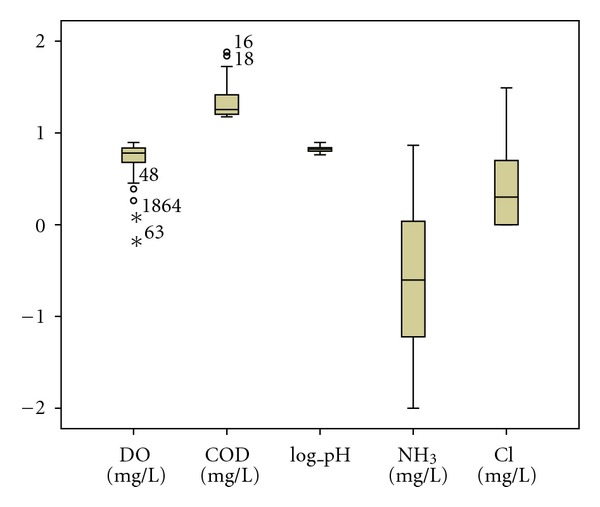
Box and whisker plot of discriminant parameters in dry season (forward and backward stepwise).

**Figure 2:**
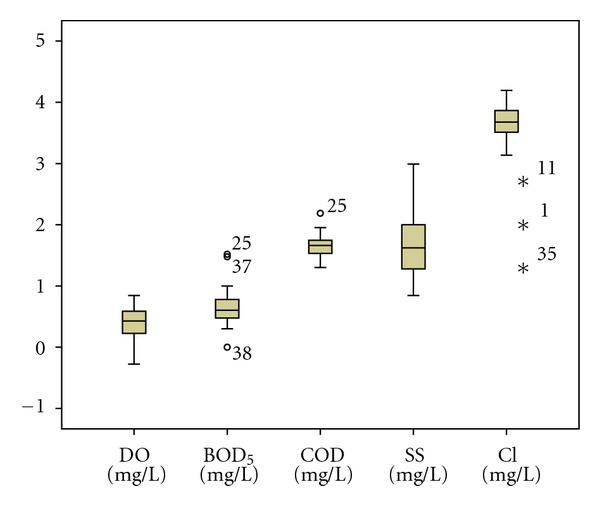
Box and whisker plot of discriminant parameters in wet season (forward stepwise).

**Figure 3:**
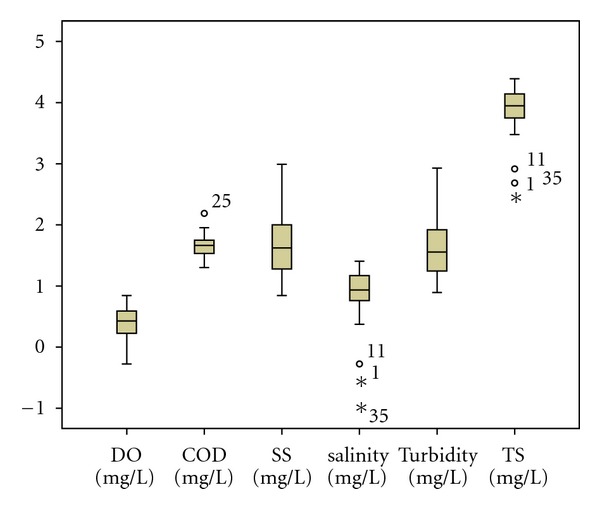
Box and whisker plot of discriminant parameters in wet season (backward stepwise).

The standard DA mode constructed discriminant functions including all the fifteen parameters under study. Forward stepwise and backward stepwise modes showed that DO, COD, pH, NH_3_, and Cl are the most significant parameters responsible for water quality variation in the dry season assigning more than 96% (*P* < 0.05) of cases correctly. In the wet season, the stepwise forward discriminant functions discriminate five variables with 68.20% (*P* < 0.05) of cases correctly. Forward stepwise mode showed that DO, BOD_5_, COD, SS, and Cl are the most significant parameters responsible for water quality variation in the wet season. However, backward stepwise DA mode produced a classification matrix of more than 82% (*P* < 0.05) correct assignations using six variables: DO, COD, salinity, turbidity, SS, and TS. The box and whisker plots of discriminating parameters identified by DA (forward stepwise and backward stepwise) for both seasons were given in Figures 1, 2 and 3.

### 3.3. Temporal Control Relationship between Variables

Partial correlation was applied to the log transformed data to estimate the correlation between BOD_5_ and COD controlling for the linear effect of NH_3_ in the dry season data and TS and DS controlling for conductivity in the wet season data. There was a strong positive correlation (*r*_*p*_ = 0.829, *P* = 0.0001) with high content of NH_3_ being associated with high level of BOD_5_ and COD and a moderate positive correlation (*r*_*p*_ = 0.614, *P* = 0.0001) with high content of conductivity associated with high level of TS and DS for dry and wet season water quality variation (Table 4).

An inspection of zero-order correlation of dry season (*r* = 0.866) and wet season (*r* = 0.993) suggests that controlling for NH_3_ and conductivity for dry and wet seasons, respectively has strong influence.

### 3.4. Temporal Water Quality Predictors

To find out the best predictor of water quality variation in the Jakara Basin, a stepwise multiple linear regression model was used. Before interpreting the result, classical assumptions of linear regressions were checked: an inspection of normal p-p plot of regression standardized residuals revealed that all the observed values fall roughly along the straight line indicating that the residuals are from normally distributed population. Moreover, the scatter plot (standardized predicted values against observed values) indicated that the relationship between the dependent variable and the predictors is linear and the residuals variances are equal or constant.

Based on the collinearity diagnostic table obtained, none of the models dimensions has conditional index about the threshold limit 30.0, none of the tolerance values is smaller than 0.10, and none of the VIF statistics is less than 10.0. This indicated that there is no multicollinearity problem among the predictors variables of the models. Since there is no multicollinearity problem between the predictors included in the dry and wet seasons samples in the final models and the classical assumptions of normality, linearity and equality of variance are all met. It is reasonably to conclude that estimated multiple linear regression models to explain water quality variation in the Jakara Basin are stable, good, and quite respectable.

#### 3.4.1. Dry Season Water Quality Predictors

Based on the stepwise method of linear regressions, seven predictor variables were found to be of significance in explaining water quality variation in dry season (Table 5). The water quality variation was explained by seven predictors, namely, DO, COD, SS, NH_3_, temperature, pH, and conductivity, other variables were excluded because they did not contribute in explaining dry season water quality variation. The obtained *R*-square of 0.976 implies that the seven predictor variables explained about 97.6% of the water quality variation in the dry season.

The ANOVA table revealed that the *F*-statistics (*F* = 381.22) was very large and the corresponding *P* value was highly significant (*P* = 0.0001) or lower than the alpha value (0.05). As depicted in Table 5, the largest beta coefficient was DO with 0.539, this means that DO makes the strongest unique contributions in explaining the variation of water quality in dry season, when the variance explained by other predictors in the model is controlled. This showed that, one standard deviation increase in the concentration of DO is followed by 0.539 standard deviation increase in the variation of water quality in the dry season. The Beta value for COD was the second highest (−0.423), followed by NH_3_ (−0.184), SS (−0.97), temperature (0.079), and conductivity (−0.052). The beta value for pH was the smallest (−0.050) and indicating that it made at least contribution in the water quality variation in the dry season.

#### 3.4.2. Wet Season Water Quality Predictors

The water quality variation in the wet season was explained by five predictor variables, namely, DO, BOD_5_, SS, TS, and Cl. The *R*-square of 0.94.2 revealed that 94.2% of the variation of water quality during wet season was explained by the mentioned five predictors. The wet season estimate of coefficient of the model is presented in Table 6. The largest beta coefficient among the parameters calibrated by stepwise regression analysis, TS, makes the strongest unique contribution in the wet season water quality variation. The beta value for DO (0.547) was the second highest, followed by Cl (0.545) and BOD_5_ (−0.292), and the least contributor was SS with −0.292.

 The ANOVA table showed that the *F*-statistics (*F*= 112.697) was very large and the corresponding *P* value is highly significant (*P* = 0.0001) or lower than the alpha value (0.05). This indicated that the slope of the estimated linear regression model is not equal to zero for both seasons, confirming that there is linear relationship between the predictors of the models. 

### 3.5. Temporal Water Quality Variation

**Figure 4:**
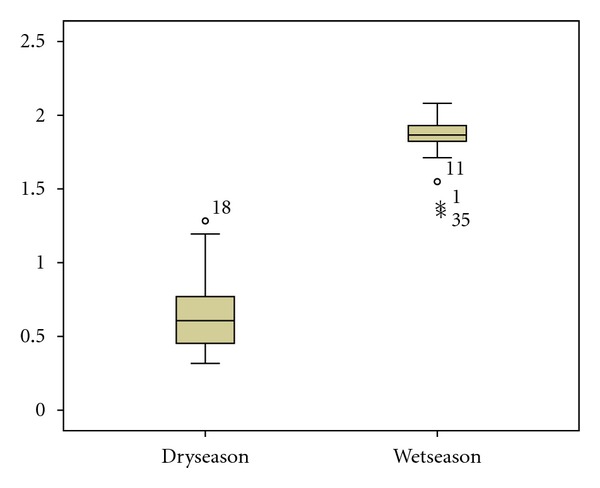
The box plot of mean of samples of dry and wet seasons.

Temporal variation of water quality was examined using repeated measure sample *t*-test, this determines whether the mean of samples obtained in the dry season differ from that of wet season samples. A quick check of the box plot shown in Figure 4 indicates that the mean of the wet season is much higher than the mean of the dry season.

Repeated measure sample *t*-test was conducted to compare means of dry and wet season samples. The null hypothesis states that there are no differences in the mean samples of river water quality in the dry and wet seasons. A preliminary assumption testing was checked for normality with no violation noted (*KS* = 0.113, *P* = 0.200), and the Q-Q plot indicated that the distribution for the dry season is normal. Although the test for normality for wet season samples did not showed a perfect normal distribution (*KS* = 0.103, *P* = 0.100), an inspection of the Q-Q plot for wet season samples show that the distribution is approaching normal. The detrended Q-Q plot showed that the data fall within −0.25 to 0.75 and −1.5 to 1.0 for dry and wet seasons, respectively, showing that there are no data that deviate from normal distribution. 

The result obtained from paired sample *t*-test revealed that there is a significant difference in the mean of dry and wet seasons samples (*M* = 1.184, *SD* = 0.277, *t* = −27.372, *P* = 0.0001). The decision is that the null hypothesis was rejected and research hypothesis was supported, this is because the mean differences obtained were rather-large and the *t*-statistics obtained was very large (*t* = −27.372) and the corresponding *P* value (0.0001) was very much smaller than the alpha of 0.05. Comparing the eta-square obtained (*η* ^2^ = 0.86) to Cohen [28] criteria (0.01 = small effect, 0.06 = moderate effect, and 0.14 large effect), the magnitude of the mean differences was large (*η* ^2^ = 0.86) showing that river water quality varies largely between the two seasons. 

## 4. Conclusion

In this study, different statistical techniques were used to assess temporal variation in surface water quality of the Jakara River Basin. DA rendered an important data reduction as it uses only five and six parameters (DO, COD, pH, NH_3_, and Cl and DO, BOD_5_, SS, salinity, turbidity, and Cl) affording more than 96% and 68% correct assignation for dry and wet seasons, respectively. Thus, DA allowed reduction in the dimensionality of the large data sets and revealing few indicator parameters responsible for large variation in water quality. Further, partial correlation analysis revealed strong and moderate partial correlation between BOD_5_ and COD, TS and DS controlling for the linear effect of HN_3_ and conductivity for the dry and wet seasons, respectively. Multiple linear regressions supported DA and identified the contribution of each variable with significant value *r* = 0.988, *R* ^2^ = 0.976 and *r* = 0.970, *R* ^2^ = 0.942 (*P* < 0.05) for dry and wet seasons, respectively. Repeated measure t-test confirmed that the surface water quality varies significantly between dry and wet season samples (*P* < 0.05). These statistical tools provided more objective interpretation of water quality variables, and, from the analyses, it is clear that DO, COD, BOD_5_, NH_3_, Cl, SS, turbidity, pH, and salinity were found to be the most abundance parameters responsible for water quality variation in the Jakara River Basin. Consequently, this study suggests that further studies in this area should be conducted to identify the sources of these parameters revealed by statistical techniques, so as to control the menace. 

## Figures and Tables

**Table 1:** Descriptive statistics of physicochemical parameters under study in mg/L except temperature, conductivity, and pH.

Parameters	Dry season					Wet season				
	Mean	SD	Variance	Skewness	Kurtosis	Mean	SD	Variance	Skewness	Kurtosis
DO	0.72	0.19	0.04	−2.50	7.08	0.41	0.24	0.06	−0.37	0.28
BOD_5_	0.22	0.33	0.11	1.59	2.76	0.61	0.29	0.09	1.20	2.89
COD	1.33	0.17	0.03	1.39	1.25	1.65	0.18	0.03	0.44	0.75
SS	1.25	0.32	0.10	−0.93	2.69	1.70	0.53	0.28	0.67	−0.13
pH	6.67	0.03	0.00	0.17	−0.44	7.14	0.02	0.00	−0.47	1.25
NH_3_	−0.56	0.74	0.54	0.06	−1.03	0.46	0.32	0.10	−1.65	5.20
Temperature	28.0	0.02	0.00	−0.42	−0.33	29.5	0.02	0.00	−0.14	0.29
Conductivity	1.96	0.71	0.51	1.85	2.84	4.10	0.47	0.22	−1.99	4.65
Salinity	−1.30	0.79	0.62	1.56	1.61	0.85	0.50	0.25	−1.99	4.62
Turbidity	1.36	0.30	0.09	−0.24	0.93	1.66	0.53	0.28	0.76	0.05
DS	1.54	0.69	0.48	1.78	2.97	3.87	0.47	0.22	−1.97	4.63
TS	1.74	0.44	0.19	2.06	5.19	3.89	0.42	0.17	−1.64	3.25
NO_3_	−0.70	0.45	0.20	−1.56	2.06	1.16	0.08	0.01	0.19	−0.57
Cl	0.42	0.38	0.15	0.74	−0.09	3.59	0.53	0.28	−2.39	6.52
PO_4_	−1.08	0.97	0.94	0.45	−1.42	1.44	0.09	0.01	−0.45	0.75

**Table 2:** Dry season classification functions for discriminant analysis (DA) of parameters in mg/L except for temperature, conductivity, and pH.

Variables	Standard mode		Forward stepwise mode		Backward stepwise mode	
	Lambda	*P* value	Lambda	*P* value	Lambda	*P* value
DO	0.623	<0.0001	0.623	<0.0001	0.623	<0.0001
BOD_5_	0.793	<0.0001				
COD	0.699	<0.0001	0.699	<0.0001	0.699	<0.0001
SS	0.994	0.508				
pH	0.893	0.004	0.893	0.004	0.893	0.004
NH_3_	0.725	<0.0001	0.725	<0.0001	0.725	<0.0001
Temperature	0.954	0.064				
Conductivity	0.867	0.001				
Salinity	0.776	<0.0001				
Turbidity	0.999	0.811				
DS	0.891	0.004				
TS	0.930	0.021				
NO_3_	0.988	0.339				
Cl	0.985	0.296	0.985	0.296	0.985	0.296
PO_4_	0.991	0.427				

Wilks' lambda	0.37		0.42		0.42	
Chi-square	381.57		46.60		46.60	
*P* level	<0.0001		<0.0001		<0.0001	

**Table 3:** Wet season classification function for discriminant analysis (DA) of parameters in mg/L except for temperature, conductivity, and pH.

Variable	Standard mode		Forward stepwise mode		Backward stepwise mode	
	Lambda	*P* value	Lambda	*P* value	Lambda	*P* value
DO	0.914	0.011	0.914	0.011	0.914	0.011
BOD_5_	0.851	0.001	0.851	0.001		
COD	0.839	0.035	0.839	0.035	0.839	0.035
SS	0.983	0.021	0.983	0.021	0.983	0.021
pH	0.962	0.482				
NH_3_	0.956	0.426				
Temperature	0.908	0.160				
Conductivity	0.902	0.141				
Salinity	0.903	0.144			0.903	0.144
Turbidity	0.878	0.084			0.878	0.084
DS	0.906	0.152				
TS	0.894	0.120			0.894	0.120
NO_3_	0.954	0.406				
Cl	0.912	0.001	0.912	0.001		
PO_4_	0.969	0.553				

Wilk's lambda	0.25		0.84		0.48	
Chi-square	1.82		1.96		88.45	
*P* level	0.01		0.03		0.01	

**Table 4:** Partial correlations of the dry and wet season variables.

Dry season					Wet season					
Control variables		BOD_5_	COD	NH_3_	Control variables		TS	DS	Conductivity	
	BOD_5_	1				TS	1			
	COD	0.866	1			DS	0.993	1		
	NH_3_	0.458	0.478	1		Conductivity	0.988	0.995	1	
NH_3_	BOD_5_	1	0.829		Conductivity	TS	1	0.614		
	COD	0.829	1			DS	0.614	1		

**Table 5:** Estimates of coefficient of the model (dry season) of parameters in mg/L except for temperature, conductivity, and pH.

	Beta unstandardized coefficient	Std. error	Beta standardized coefficient	*t*-value	*P* value
(Constant)	62.793	6.003		10.459	0.000
DO	4.184	0.191	0.539	21.878	0.000
COD	−0.427	0.034	−0.423	−12.645	0.000
SS	−0.076	0.015	−0.097	−4.958	0.000
NH_3_	−1.529	0.292	−0.184	−5.244	0.000
Temperature	0.629	0.175	0.079	3.595	0.001
pH	−1.266	0.492	−0.050	−2.571	0.012
Conductivity	0	0	−0.052	−2.491	0.015

*R* = 0.988; *R* ^2^ = 0.976; Adj. *R* ^2^ = 0.973.

**Table 6:** Estimates of coefficients of the model (wet season) of parameters in mg/L.

	Beta unstandardized coefficient	Std. error	Beta standardized coefficient	*t*-value	*P* value
(Constant)	40.689	7.211		5.642	0.000
DO	23.952	2.058	0.547	11.639	0.000
BOD_5_	−17.866	1.654	−0.491	−10.805	0.000
SS	−5.825	0.953	−0.292	−6.11	0.000
TS	16.979	5.225	0.668	3.25	0.003
Cl	−10.995	4.078	−0.545	−2.696	0.011

*R* = 0.970; *R* ^2^ = 0.942; Adj. *R* ^2^ = 0.933.
